# Real-time fidelity assessment of fluorescence molecular imaging without reference images

**DOI:** 10.1117/1.JBO.31.6.066001

**Published:** 2026-06-03

**Authors:** Elena Kriukova, Sarah Glasl, Vasilis Ntziachristos, Dimitris Gorpas

**Affiliations:** a Technical University of Munich, Central Institute for Translational Cancer Research (TranslaTUM), Information and Technology, School of Medicine and Health & School of Computation, Chair of Biological Imaging, Munich, Germany; b Helmholtz Zentrum München, Institute of Biological and Medical Imaging, Bioengineering Center, Neuherberg, Germany; c University of Groningen, University Medical Center Groningen, Department of Gastroenterology and Hepatology, Groningen, The Netherlands

**Keywords:** fluorescence molecular imaging, no-reference image fidelity assessment, quality control, standardization

## Abstract

**Significance:**

Fluorescence molecular imaging (FMI) has been approved for surgical guidance in several procedures. However, its interpretation still requires substantial clinician expertise due to variable signal levels. For FMI to gain wider clinical acceptance, image quality should be assessed automatically, thereby prompting clinicians to retake low-quality images when necessary. Yet, no user-friendly fidelity assessment tools currently address this need.

**Aim:**

Herein, we introduce a real-time, frame-by-frame FMI fidelity assessment method, termed no-reference fidelity assessment of fluorescence molecular imaging (NRFA-FMI).

**Approach:**

NRFA-FMI quantifies image fidelity using a combination of histogram-based and textural metrics. A central advantage of NRFA-FMI is that it operates without reference images, making it particularly suitable for clinical FMI workflows.

**Results:**

We show that NRFA-FMI outperforms two state-of-the-art no-reference image fidelity assessment methods, the blind/referenceless image spatial quality evaluator (BRISQUE) and the natural image quality evaluator (NIQE).

**Conclusions:**

The introduction of NRFA-FMI enables real-time FMI fidelity assessment, an essential step toward reducing data misinterpretation and preventing incorrect diagnosis. This approach provides clinicians with an intuitive, automated indicator of image reliability, supporting more consistent and accurate intraoperative decision-making.

## 1 Introduction

Fluorescence molecular imaging (FMI) can visualize fluorescent biomarkers of disease, allowing monitoring of specific dysregulated molecular processes in real time. Thus, FMI is often referred to as a “red-flag” technology and is increasingly considered for enabling surgical guidance or diagnosis for various medical specialties, including oncology. For example, more cancer lesions can be detected using fluorescent labels compared with inspection with white-light imaging only.^1^^,^^2^ However, FMI widespread clinical adoption is unlikely until there is a robust method for ensuring high-fidelity image acquisition, that is, accurate representations of fluorochrome biodistributions in tissues.^3^^,^^4^

Fidelity assessment of images is particularly critical as diagnoses and interventions are highly dependent on image quality, which can be dynamically affected by many factors, such as noise, low tumor-to-background ratios (TBRs), specular reflectance, and intrinsic tissue fluorescence.^3^^,^^5^ Advanced image processing tools are also needed to remove barriers to FMI use by providing simple and easy-to-understand user prompts. These prompts, for example, include the quantification of fidelity metrics that are representative of the dynamic range, histogram, and texture features of each image. Therefore, the development of such a tool for real-time user-independent fidelity assessment of FMI readouts will accelerate the clinical translation of the technology.

Currently, fidelity of fluorescence images is frequently assessed using signal intensity, signal-to-noise ratio (SNR), contrast-to-noise ratio (CNR), and TBR.^4^^,^^5^ These metrics require manual selection of regions of interest (ROIs), which prevents real-time applications and leads to bias and irreproducibility.^6^^–^^8^ Moreover, we recently showed that metrics like SNR and contrast are also strongly affected by the choice of formulas used to define them.^9^ The detected signal intensities may also be affected by additional factors, such as the specificity of the fluorescent agent, the tissue optical properties, and the technical specifications and performance of the imaging system.^10^^,^^11^

To address the critical issue of subjectivity in image fidelity assessment, new image processing techniques aimed at achieving high-fidelity fluorescence imaging (HiFFI)^3^ have been developed. One such technique is the quantitative fluorescence single-snapshot of optical properties (qF-SSOP) imaging, which estimates optical properties of the imaged sample and corrects distortions in fluorescence data.^12^ This method has been used to enhance fidelity of phantom and hand vasculature images, providing real-time optical property corrected fluorescence images with less than 5% error. However, one limitation of qF-SSOP is that the image background and edge ripples are not considered when assessing fidelity, often leading to inconsistent results across the entire field of view. Another method, called spatially adaptive impulse response correction (SAIRC), enhanced the resolution, contrast, and sharpness of fluorescence images from phantoms and animal models by means of an iterative deconvolution procedure.^13^ However, both these methods require reference images and use algorithmic approaches that hinder real-time operation and visualization.^12^^,^^13^

No-reference image quality assessment (NR-IQA) methods are widely used in digital image processing and video streaming applications to assess the fidelity of natural images.^14^^,^^15^ Some NR-IQA methods, such as the blind/referenceless image spatial quality evaluator (BRISQUE)^15^ or the natural image quality evaluator (NIQE),^16^ have also been tested on fluorescence images with limited success. These methods were developed for broadband reflectance images and estimate natural statistics from the images, which is not always a valid approach for fluorescence images that frequently lack natural context.^17^^–^^19^

Therefore, the lack of real-time no-reference image fidelity assessment methods for FMI lead to subjective interpretations of the readouts and prevents the effective “red-flag” operation of the technology. Here, we propose a new method, termed no-reference fidelity assessment of fluorescence molecular imaging (NRFA-FMI), that (1) avoids subjective definition of signal and background ROIs by avoiding dependance on SNR and CNR quantification, (2) is developed based on FMI data recorded from a multiparametric fluorescence standard,^20^ and (3) does not require any reference images. Although it is not the first real-time method for image fidelity assessment, NRFA-FMI is, to our knowledge, the first method specifically designed for FMI applications. As such, it addresses the longstanding need for robust image fidelity assessment in FMI.

The NRFA-FMI method combines histogram and textural statistics to assign an image quality value.^21^^,^^22^ High-fidelity images have quality values that comply with two criteria; the texture criterion, indicating the minimum detectable signal according to the AAPM guidelines,^4^ and the histogram criterion defining the limit of saturated pixels in an FMI image. Images not meeting both criteria can either be discarded or labeled as low fidelity.

The efficacy of the proposed NRFA-FMI method was experimentally demonstrated through four simulated scenarios that affect image quality during FMI. These scenarios include variability in FMI acquisition settings, inhomogeneity in the illumination spatial distribution, and motion. Both still images and video frames were evaluated to showcase the real-time frame-by-frame application of the NRFA-FMI method. Finally, the performance of the method was compared against two state-of-the-art image quality assessment methods, i.e., BRISQUE and NIQE, that have been applied in fluorescence microscopy^17^^,^^18^ and imaging.^19^

## 2 Materials and Methods

### 2.1 FMI System

To obtain the fluorescence imaging data for the current study, we used an FMI system that has been previously reported by our group.^23^ Fluorescence was induced by a 750-nm diode laser (FLX-750-1500 M-100-9 MM, Frankfurt Laser Company, Friedrichsdorf, Germany) and detected by 14-bit iXon electron multiplying charge-coupled device (iXon3 EMCCD, DV897DCS-BV, Andor Technology, Belfast, Northern Ireland). At the same time, color imaging was enabled by a 250-W halogen lamp (KL-2500 LCD, Schott AG, Mainz, Germany) and detected by a 12-bit color charge-coupled device (CCD, pixelfly qe, PCO AG, Kelheim, Germany). A combination of a dichroic mirror (700DCXXR, AHF analysentechnik AG, Tubingen, Germany) and filters (ET810/90 for fluorescence and ET700SP-2P for color imaging, Chroma Technology, Rockingham, Vermont, US) separated the optical signal collected by the objective into the fluorescence and color channels, as described in our previous work,^23^^,^^24^ enabling concurrent fluorescence and color imaging. In this study, only the images acquired using the fluorescence channel were assessed for fidelity.

### 2.2 Fluorescence Standard

**Fig. 1:**
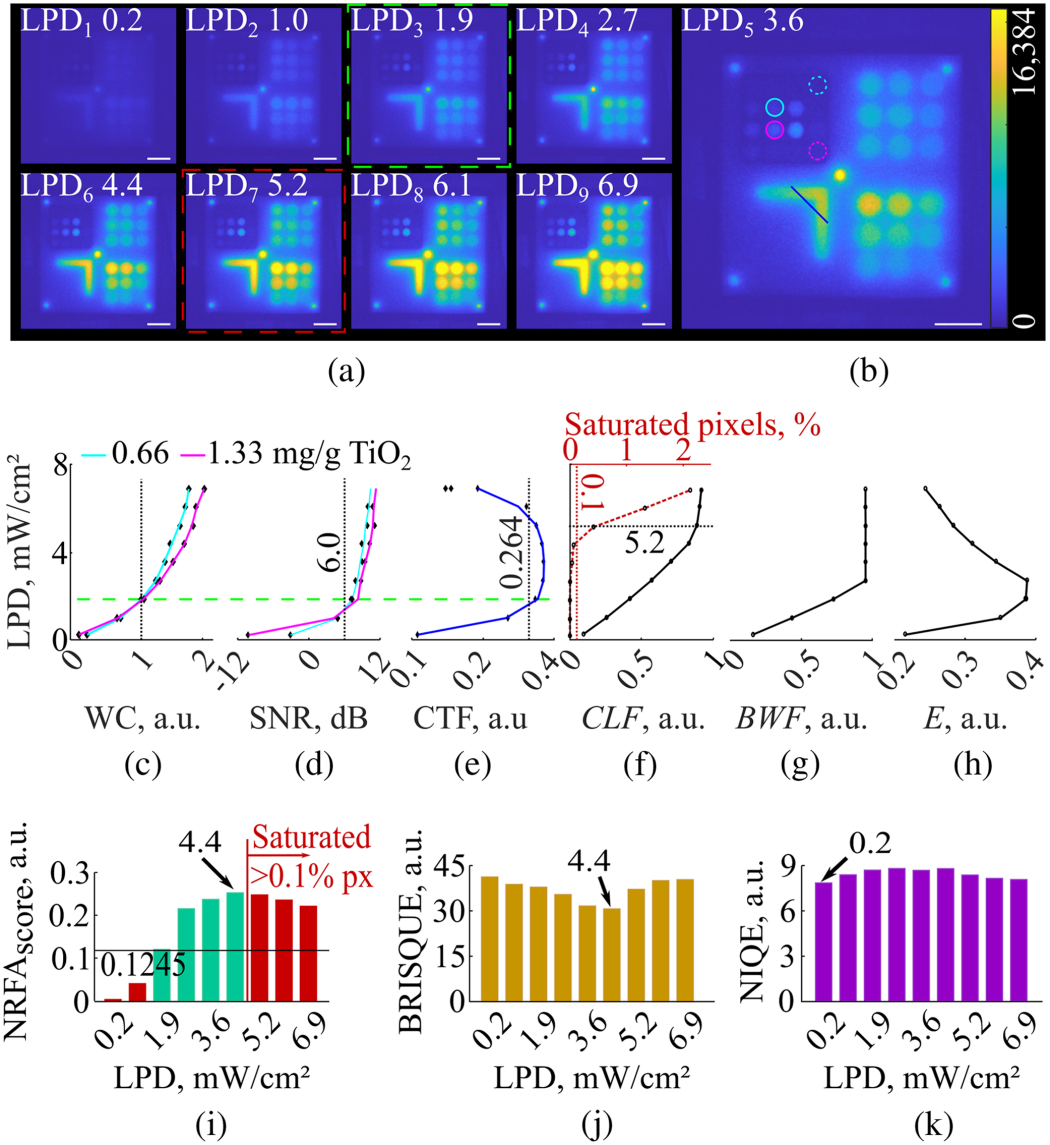
Definition of the no-reference fidelity assessment of fluorescence molecular imaging (NFRA-FMI) criteria. (a) Images of the fluorescence standard recorded with increasing laser power density (LPD in $\mathrm{mW}/{\mathrm{cm}}^{2}$). The images within the green and red dotted boxes were used for the NRFA-FMI texture and histogram criteria, correspondingly. The scale bars correspond to 2 cm. (b) The image acquired under LPD $3.6\;\mathrm{mW}/{\mathrm{cm}}^{2}$, with cyan and magenta circles marking the regions of interest (ROIs) used for the estimation of the signal-to-noise ratio (SNR) and Weber contrast (WC). The contrast transfer function (CTF) was estimated along the blue line. The scale bar corresponds to 2 cm. (c) WC, (d) SNR, and (e) CTF for all images in panels (a) and (b) with the corresponding thresholds (black lines). The dashed green line corresponds to the image of panel (a) used for the texture criterion. (f) The chromatic level factor (CLF), (g) bandwidth factor (BWF), and (h) energy (E) for all images of panels (a) and (b). The dashed black and red lines in panel (f) indicate the image in panel (a) used for the histogram criterion. (i) The ${\mathrm{NRFA}}_{\text{score}}$ for all nine images. The green bars indicate high-fidelity images, whereas images with low fidelity are marked in red. The image with highest fidelity is the one acquired with $4.4\;\mathrm{mW}/{\mathrm{cm}}^{2}$ excitation. (j) The blind/referenceless image spatial quality evaluator (BRISQUE) scores. The highest fidelity is under $4.4\;\mathrm{mW}/{\mathrm{cm}}^{2}$ excitation. (k) The naturalness image quality evaluator (NIQE) scores. The highest fidelity is under $0.2\;\mathrm{mW}/{\mathrm{cm}}^{2}$ excitation.

A detailed description of the fluorescence standard used in this study is provided in our previous works.^20^^,^^24^^,^^25^ Briefly, the main constituents of the fluorescence standard were alcohol-soluble nigrosin (Sigma Aldrich, St. Louis, Missouri, US) and bovine hemin (≥90% pure; Sigma Aldrich, St. Louis, Missouri, US) for mimicking absorption and titanium dioxide (${\mathrm{TiO}}_{2}$) nanoparticles (Titanium IV Oxide; Sigma Aldrich, St. Louis, Missouri, US) for mimicking scattering. Organic quantum dots (Qdot 800 ITK, Thermofisher Scientific, Waltham, Massachusetts, US) were used as the fluorescent dye. The fluorescence standard [see Fig. 1(b)] consists of four quadrants: (1) “dynamic range and light leakage” on the top left, (2) “sensitivity as a function of optical properties” on the top right, (3) “sensitivity as a function of depth” on the bottom right, and (4) “resolution” on the bottom left.^20^ Herein, we only used the “Dynamic range and light leakage” and the “Resolution” quadrants for the definition of the NRFA-FMI criteria.

### 2.3 Animal Models

Two 8-week-old female athymic nude Foxn1nu mice (Envigo, Germany) were used in this study. Each mouse was subcutaneously injected with 4T1 tumor cells ($1\times 10^{6}$ 4T1 murine breast cancer cells in 20  μL of a phosphate buffer saline suspension). Seven days after 4T1 cell administration, one mouse was intravenously injected with 2 nmol IntegriSense 750 (Perkin Elmer, Waltham, Massachusetts, US) and the second one with 2 nmol Angiosense 750 (Perkin Elmer, Waltham, Massachusetts, US). The mice were then imaged with the FMI system 24 h posttracer injection under isoflurane anesthesia. Following the imaging procedures, both mice were sacrificed.

All experiments were performed according to the Committee on Animal Health and Care of Upper Bavaria, Germany. The mice were maintained in an individually ventilated cage system (Tecniplast, Germany) at 22°C ambient temperature, ∼50% relative humidity, and regular 12 h day/night cycle in our specific-pathogen-free (SPF) mouse facility.

### 2.4 NRFA-FMI Score

The NRFA-FMI method is based on the estimation of a fidelity score (i.e., the ${\mathrm{NRFA}}_{\text{score}}$) defined as

$${\mathrm{NRFA}}_{\text{score}}=\mathrm{BWF}\cdot \mathrm{CLF}\cdot E,$$ (1)

where BWF is the bandwidth factor,^22^ CLF is the chromatic level factor,^22^ and E is the image Energy.^21^

The BWF corresponds to the normalized bandwidth of a single grayscale fluorescence image and is calculated as^22^

$$\mathrm{BWF}=(R-L+1)/2^{n},$$ (2)

where n is the camera bit depth and R and L are, the right and left boundary bins, respectively, of the image histogram. BWF represents the bandwidth of the FMI images normalized to the bit depth of the camera, and its maximum value possible is 1.

Similarly, the CLF represents the complex of chromatic level of the fluorescence image and is calculated as^22^

$$\mathrm{CLF}=\overset{\left(2^{n}-1\right)}{\underset{0}{\sum }}p(n)/2^{n},$$ (3)

where n is the bit depth and p(n) is the distribution of the histogram bins and is equal to 0 when no pixels exist for the n bin or 1 when there is one or more pixels. Thus, similar to BWF and CLF would also reach a maximum value of 1.

In contrast to BWF and CLF, which are both metrics related to the histogram of the FMI image, E is associated with the image texture and is derived from the gray-level co-occurrence matrices (GLCM).^21^ E is the sum of squared elements in the GLCM and is also known as uniformity or the angular second moment. This metric is calculated from the formula^21^^:^

$$E=\underset{i,j}{\sum }u(i,j{)}^{2},$$ (4)

where u(i,j) is the (i,j) element of the normalized GLCM as computed by the function “graycomatrix” of MATLAB (Mathworks Inc., Natick, Massachusetts, US). The ${\mathrm{NRFA}}_{\text{score}}$ for each FMI frame is then quantified according to (1) and its values range between 0 and 1.

### 2.5 BRISQUE and NIQE Methods

The proposed NRFA-FMI method was compared against two established and independent NR-IQA methods that both depend on features of natural scene statistics. The first method, BRISQUE,^15^ was calculated using the “brisque” function of MATLAB. This method is typically only used for specific distortion types. The second method, NIQE,^16^ is considered to be a more general method than BRISQUE and was calculated using the “niqe” function of MATLAB. For both models, the calculated scores can be of any value with the lowest values considered to have highest fidelities.

### 2.6 Definition and Validation of the NRFA-FMI Method

#### 2.6.1 Experiment 1 (Expt. 1) – definition of NRFA-FMI criteria

To define the NRFA-FMI texture and histogram criteria and the corresponding threshold values, we imaged the fluorescence standard under constant EMCCD gain and exposure time and with incident laser power density ranging from 0.2 to $6.9\;\mathrm{mW}/{\mathrm{cm}}^{2}$ [Fig. 1(a)]. All acquisition settings for Expt. 1 are summarized in Table 1, whereas Table 2 shows the laser power densities applied during Expt. 1.

**Table 1:** Acquisition settings for Expt. 1 to 4.

	Power density ($\mathrm{mW}/{\mathrm{cm}}^{2}$)	EMCCD camera gain	Exposure time (ms)
Expt. 1	0.2 to 6.9^a^	300	100
Expt. 2	1.0 to 10.1^a^	300	80
Expt. 3	10.1	2/40/80	60/80/100/150/200/250/300
Expt. 4	10.1	40	60

Expt, experiment; EMCCD, electron multiplying charge-coupled device.

a See Table 2 for full range of values.

**Table 2:** Laser power on the imaged plane for Expt. 1 to 4.

	Power density ($\mathrm{mW}/{\mathrm{cm}}^{2}$)
Expt. 1	0.2; 1.0; 1.9; 2.7; 3.6; 4.4; 5.2; 6.1; 6.9
Expt. 2	1.0; 1.9; 2.7; 3.6; 4.4; 5.2; 6.1; 6.9; 7.7; 8.5; 9.3; 10.1
Expt. 3	10.1
Expt. 4	10.1

Expt, experiment.

##### 2.6.1.1 Definition of the NRFA-FMI texture criterion

The definition of the NRFA-FMI texture criterion was based on the SNR, the Weber contrast (WC), and the contrast transfer function (CTF) calculated from the formulas in Table 3.

**Table 3:** Three metrics used for the definition of the no-reference fidelity assessment of fluorescence molecular imaging (NRFA-FMI) texture criterion.

Metric	Formula	Threshold	Ref.
Signal-to-noise ratio (SNR)	$\mathrm{SNR}=\frac{S(C)-S(0)}{\sigma (0)}$	6 dB^20^	4
Weber contrast (WC)	$\mathrm{WC}=\frac{S(C)-S(0)}{S(0)}$	1^20^	26
Contrast transfer function (CTF)	$\mathrm{CTF}=\frac{\max(I)-\min\left(I\right)}{\max(I)+\min(I)}$	26.4%^23^	24

S(C), mean pixel intensity of well with concentration, C; S(0), mean pixel intensity of background; σ(0), standard deviation of background pixel intensity; max(I), min(I), maximum and minimum pixel intensity values.

The SNR and WC were quantified within the cyan and magenta circles shown in Fig. 1(b). The solid circles indicate 2 wells with different scattering by the same Qdot concentrations (i.e., 0.66 g and 1.33  mg/g ${\mathrm{TiO}}_{2}$ for the cyan and magenta circles correspondingly; 10 nM Qdot), whereas the dashed circles indicate background ROIs (0 nM QDot^20^). The third metric, i.e., CTF, was quantified from the intensity profile along the blue line marked in Fig. 1(b).^24^

The NRFA-FMI texture criterion is, therefore, defined as the ${\mathrm{NRFA}}_{\text{score}}$ of the fluorescence image under the lower incident laser power density, in which all three metrics –SNR, contrast, and CTF– exceed their respective threshold values shown in Table 3. This criterion ensures that all images meeting this definition maintain both sensitivity and sharpness.

##### 2.6.1.2 Estimation of NRFA-FMI histogram criterion

The NRFA-FMI histogram criterion was determined by the CLF and the image histogram. As the laser power density increased (see Table 2), the histogram shifted to the right, gradually increasing the CLF value. When the full dynamic range of the camera was reached, the CLF became constant. Based on Ref. 3, if the laser power density continued to increase, the CLF would gradually decrease as more pixels will get saturated.

To establish the NRFA-FMI histogram criterion, we first estimated the CLF values for all fluorescence images and identified the point where CLF ceases to increase. Because CLF is highly dependent on image information that shapes the histogram, the NRFA-FMI histogram criterion was defined as the percentage of saturated pixels at this point. Any FMI image exceeding this saturation level is expected to lack essential information and can be considered of low fidelity.

#### 2.6.2 Expt. 2 – validation of NRFA-FMI

To validate the NRFA-FMI method, we imaged *in vivo* a mouse injected with IntegriSense 750 under the acquisition settings in Table 1 and the laser power of Table 3. For all images, the NRFA-FMI, BRISQUE, and NIQE scores were calculated. The image histograms with the highest quality were then compared in terms of saturation and distribution.

#### 2.6.3 Expt. 3. – NRFA-FMI under different acquisition settings

The incident excitation power is only one of the parameters that influence the fidelity of FMI recordings. The camera gain and exposure time can also affect the quality of the acquired images, as well as the yield of the used tracer. To investigate the performance of the NRFA-FMI method when gain and exposure time were changed, we imaged *in vivo* the mouse injected with Angiosense 750, as summarized in Table 1. Similar to Expt. 2, the NRFA-FMI, BRISQUE, and NIQE scores were calculated and the histograms of the resulted images were compared in terms of saturation and distribution.

#### 2.6.4 Expt. 4 – NRFA-FMI for a moving target

Motion within the FMI system’s field of view (FoV) is also affecting the image fidelity, especially due to nonuniform illumination. To investigate how the NRFA-FMI method performed for a moving target within a nonuniform illumination field, we recorded a video of the mouse injected with Angiosense 750 as it was moved within the FoV, whereas it was anesthetized on a heating pad. The NRFA-FMI, BRISQUE, and NIQE scores were calculated for all frames to assess each method’s performance in a realistic scenario. The three methods were also compared in terms of time required for quality assessment to evaluate their real-time potential.

### 2.7 Data Acquisition and Processing

Data acquisition and control of the FMI system was done by a software previously developed by our group.^23^ All data processing was done in MATLAB.

## 3 Results

### 3.1 Expt. 1 – Definition of NRFA-FMI Criteria

To define the NRFA-FMI criteria, we acquired nine images of the fluorescence standard under the acquisition settings of Table 1. As expected, the pixel intensities in the acquired images increase with respect to laser power [Figs. 1(a)–1(b)].

The SNR, WC, and CTF were quantified using the formulas of Table 3, and the ROIs shown in Fig. 1(b) [Figs. 1(c)–1(e)]. The lowest laser power density associated with the minimum image SNR, WC, and CTF values was $1.9\;\mathrm{mW}/{\mathrm{cm}}^{2}$ [Figs. 1(c)–1(e), green dashed line]. Using the corresponding image [Fig. 1(a), green dotted box], the NRFA-FMI texture criterion was, then, calculated from (1) as ${\mathrm{NRFA}}_{\text{score}}=0.1245$.

The CLF values for the nine fluorescence images are shown in Fig. 1(f), where a CLF increase and transition to constant values is shown (see Sec. 2.6). The NRFA-FMI histogram criterion only requires the point, in which the CLF transitions from increasing to constant values, which occurred at $5.2\;\mathrm{mW}/{\mathrm{cm}}^{2}$ laser power density [Fig. 1(f)]. In the corresponding image [Fig. 1(a), red dotted box], >0.1% pixels were saturated. Therefore, the NRFA-FMI histogram criterion was determined to be 0.1% saturated pixels. Any image with higher number of saturated pixels should be discarded or labelled as low fidelity.

Figures 1(g) and 1(h) show the BWF and E, respectively. In Fig. 1(g), we observe that the BWF increases with laser power density. However, when the first pixel got saturated, BWF transitioned to a constant value. If the incident laser power density was further increased, we would observe a decrease in the BWF value according to Eq. (2). On the other hand, the E [Fig. 1(h)] indicates the uniformity of each image and its value increases as the pixel signal becomes higher than the noise. Once the first pixel gets saturated, E starts decreasing due to the intensity differences between saturated and nonsaturated pixels. Combining CLF, BWF, and E in Eq. (1), the ${\mathrm{NRFA}}_{\text{score}}$ values for all nine images were calculated and are shown in Fig. 1(i). The highest ${\mathrm{NRFA}}_{\text{score}}$ among all images meeting both criteria is achieved at a laser power density equal to $4.4\;\mathrm{mW}/{\mathrm{cm}}^{2}$.

Expt. 1 demonstrates the complementary roles of the texture and histogram criteria in NRFA-FMI. Although the texture criterion, derived from the lowest excitation condition satisfying SNR, Weber contrast, and CTF thresholds, establishes a lower bound for sensitivity and spatial sharpness, the histogram criterion independently identifies fidelity loss due to saturation by detecting the point at which CLF no longer increases and pixels begin to saturate [Figs. 1(f)–1(h)].

For reference, the BRISQUE [Fig. 1(j)] and the NIQE [Fig. 1(k)] scores were both quantified as described in Sec. 2.5. For BRISQUE, the image with the highest quality coincides with the result of the NRFA-FMI method. However, the NIQE converges to the image with $0.2\;\mathrm{mW}/{\mathrm{cm}}^{2}$ laser power density, where the pixel intensities were very close to the noise level.

### 3.2 Expt. 2 – Validation of NRFA-FMI

**Fig. 2:**
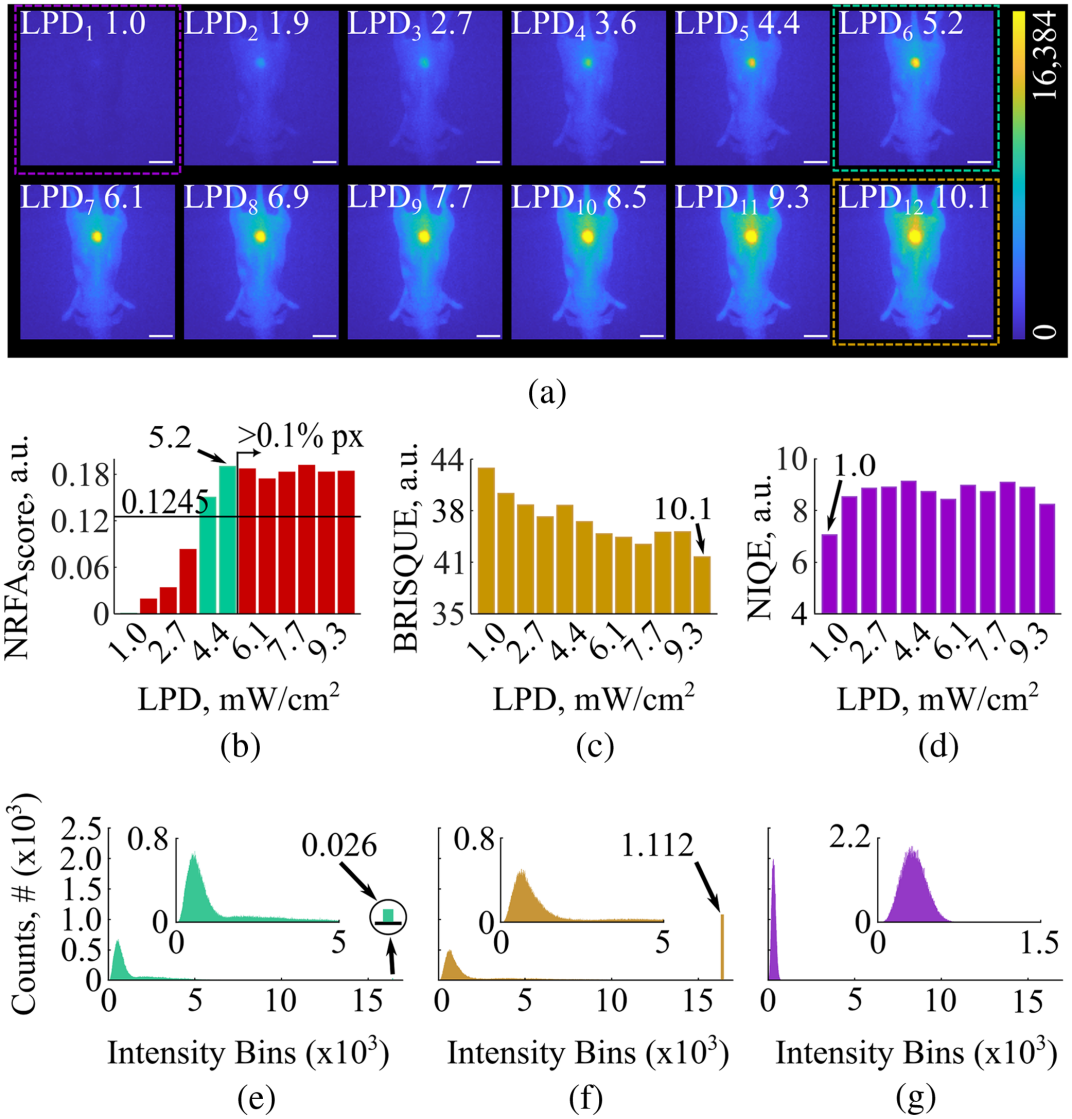
Fidelity assessment of fluorescent images acquired *in vivo* of a 4T1 tumor mouse model injected with integriSense 750, using the nonreference fidelity assessment (NRFA-FMI), blind/ referenceless image spatial quality evaluator (BRISQUE) and naturalness image quality evaluator (NIQE) methods. (a) Fluorescence images recorded with increasing laser power density. The green, orange, and magenta dashed boxes represent the images of highest fidelity as derived by the NRFA-FMI, BRISQUE, and NIQE methods. The scale bars correspond to 10 cm. (b) The ${\mathrm{NRFA}}_{\text{score}}$ values for all 12 images shown in panel (a). All images not complying with the two NRFA-FMI criteria are marked with red color, whereas the green bars represent the two images meeting both criteria. (c) The BRISQUE scores for all 12 images of panel (a). The image with the lowest score was acquired under $10.1\;\mathrm{mW}/{\mathrm{cm}}^{2}$ excitation. (d) The NIQUE scores for all 12 images of panel (a). The image with the lowest score was acquired under $1.0\;\mathrm{mW}/{\mathrm{cm}}^{2}$ excitation. (e) The histogram of the image with the highest ${\mathrm{NRFA}}_{\text{score}}$. The region of the dynamic range where most pixels are concentrated is shown in the inset. Only 26 pixels were saturated. (f) The histogram of the image with the lowest BRISQUE score. The region of the dynamic range where most pixels are concentrated is shown in the inset. 1112 pixels were saturated. (g) The histogram of the image with the lowest NIQE score. The region of the dynamic range where most pixels are concentrated is shown in the inset. Although no pixels were saturated, the distribution of the pixel intensity values lies within the lower end of the camera’s dynamic range (i.e., pixel intensity values were at the noise level).

In Expt. 2, the NRFA-FMI method was compared with the BRISQUE and NIQE ones by imaging a mouse bearing a subcutaneous 4T1 tumor *in vivo*, as described in Sec. 2.6. The acquired images are shown in Fig. 2(a). Similar to the results of Expt. 1, the increase in the laser power density resulted in higher pixel intensity values, with a growing number of pixels reaching saturation.

The bar-plots of Figs. 2(b)–2(d) show the fidelity scores for all 12 images of Fig. 2(a) using the three image fidelity assessment methods. In contrast to BRISQUE and NIQE, the proposed NRFA-FMI model discards 10 out of the 12 acquired images falling beyond the critical values for the two criteria shown in Fig. 2(b). The image acquired under $5.2\;\mathrm{mW}/{\mathrm{cm}}^{2}$ laser power density had the highest ${\mathrm{NRFA}}_{\text{score}}$, or in other words, quality [Fig. 2(a), green dotted box]. The histogram of this image is shown in Fig. 2(e). As expected from the fluorescence images, most pixels are concentrated towards the lower end of the camera’s dynamic range, corresponding to the nonfluorescent background. Importantly, only 26 pixels are saturated in this image, which correspond to 0.01% of the total pixel number (512×512  pixels), and is much lower than the NRFA-FMI histogram criterion of 0.1% saturated pixels.

Conversely, application of the BRISQUE method indicates that the best quality image is the one acquired under the maximum laser power density of $10.1\;\mathrm{mW}/{\mathrm{cm}}^{2}$, as shown in Fig. 2(c) (dashed orange box). Although most of the pixels in the image with the highest fidelity according to BRISQUE are concentrated toward the lower end of the camera’s dynamic range [similar to the histogram of Fig. 2(e)], there are 1112 saturated pixels [Fig. 2(f)]. This number corresponds to 0.42% of the total pixel number and is more than 4 times higher than the NRFA-FMI histogram criterion critical value of 0.1% saturated pixels. Finally, the NIQE method identifies the image under $1.0\;\mathrm{mW}/{\mathrm{cm}}^{2}$ laser excitation as the one with the highest fidelity [Fig. 2(d), dashed magenta box]. Although no saturated pixels exist in this image, it is obvious from both the visual inspection of the image in Fig. 2(a) and the histogram of Fig. 2(g) that no fluorescence contrast exists and all the pixels have intensity values at the noise level.

Therefore, NRFA-FMI does not only converge at the image with optimal balance between histogram distribution and saturated pixels, but it can also identify all the high-fidelity frames, whereas it discards poor quality images. By contrast, the score values provided by BRISQUE and NIQE do not discard any frames and identification of the image with highest fidelity simply corresponds to the lowest score.

### 3.3 Expt. 3 – NRFA-FMI under Different Acquisition Settings

**Fig. 3:**
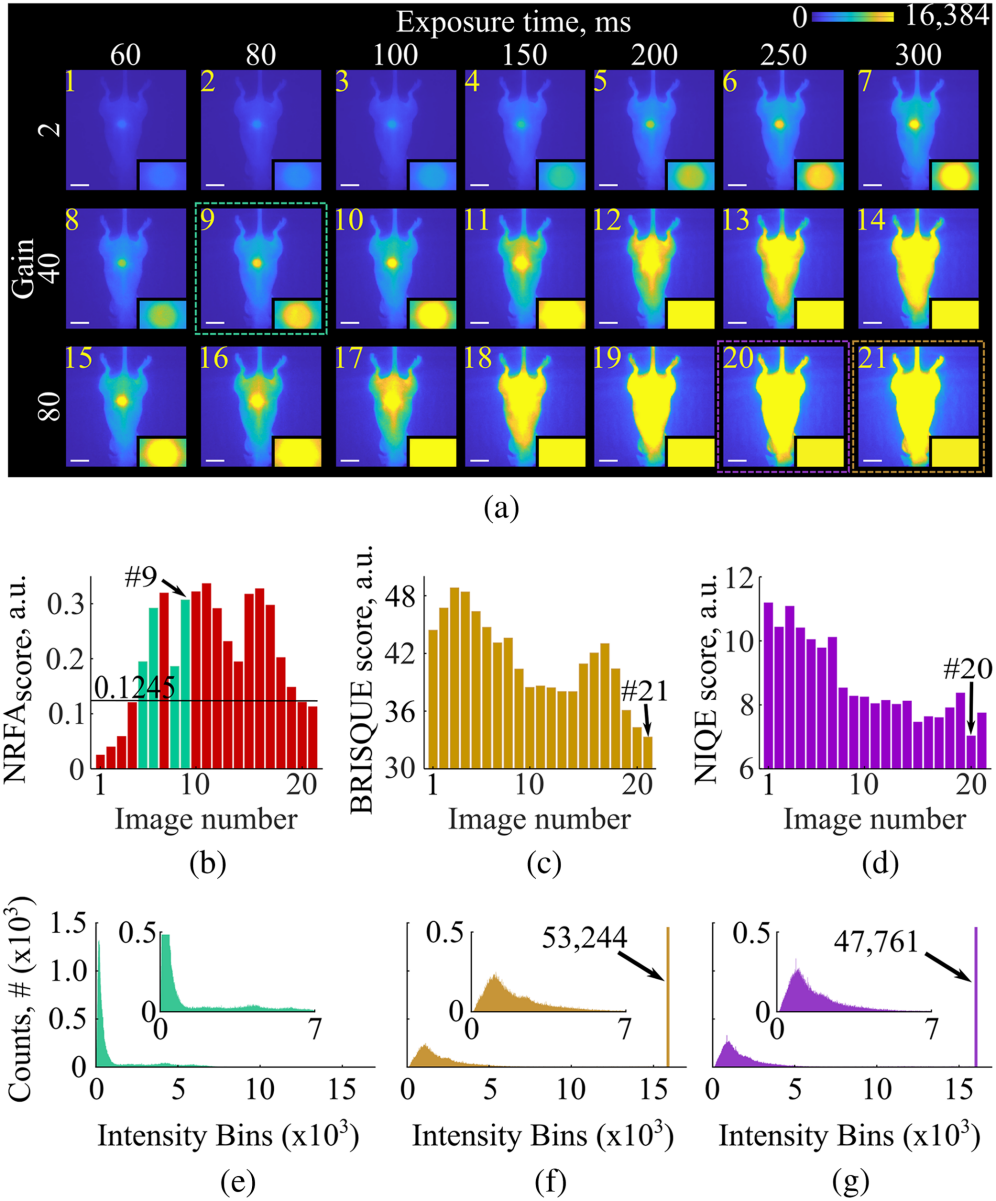
Fidelity assessment of fluorescent images acquired *in vivo* of 4T1 tumor mouse model, injected with angiosense 750, using the nonreference fidelity assessment (NRFA-FMI), blind/referenceless image spatial quality evaluator (BRISQUE), and naturalness image quality evaluator (NIQE) methods. (a) Fluorescence images recorded with various gain values and exposure times. The green, orange, and magenta dashed boxes represent the images of highest fidelity as derived by the NRFA-FMI, BRISQUE, and NIQE methods. The scale bars correspond to 2 cm. (b) The ${\mathrm{NRFA}}_{\text{score}}$ values for all 21 images shown in panel (a). All images not complying with the NRFA-FMI texture and histogram criteria are marked with red color, whereas the four green bars indicate the images meeting both criteria. (c) The BRISQUE scores for all 21 images of panel (a). (d) The NIQE scores for all 21 images of panel (a). (e) The histogram of the image with the highest ${\mathrm{NRFA}}_{\text{score}}$. The region of the dynamic range where most pixels are concentrated is shown in the inset. Only 1 pixel was saturated. (f) The histogram of the image with the lowest BRISQUE score. The region of the dynamic range where most pixels are concentrated is shown in the inset. 53,244 pixels were saturated. (g) The histogram of the image with the lowest NIQE score. The region where most pixels are concentrated is shown in the inset. 47,761 pixels were saturated.

Figure 3(a) shows 21 images recorded *in vivo* from a mouse with a 4T1 subcutaneous tumor following the administration of Angiosense 750 by using the acquisition settings of Expt. 3 (see Sec. 2.6 and Table 1). Insets in each image show a zoomed-in image of the tumor area, clearly illustrating the variation in pixel intensities with different gain and exposure time values. The calculated ${\mathrm{NRFA}}_{\text{score}}$ values are graphically presented in Fig. 3(b). Interestingly, only four images have an ${\mathrm{NRFA}}_{\text{score}}$ complying to the two NRFA-FMI criteria [green bars in Fig. 3(b)], whereas all the other images can be discarded as low-fidelity images [red bars in Fig. 3(b)]. Out of the four images assessed as high fidelity, image number 9 [Fig. 3(a), green dotted box] has the highest ${\mathrm{NRFA}}_{\text{score}}$, which is corroborated by visual assessment as the image shows maximum signal contrast balanced with minimal saturation.

Moreover, the distribution of the ${\mathrm{NRFA}}_{\text{score}}$ values can be linked to the combination of the camera’s gain and exposure time. Lower gain and exposure time result in low values in the ${\mathrm{NRFA}}_{\text{score}}$, whereas as these acquisition settings increase the ${\mathrm{NRFA}}_{\text{score}}$ values also increase, until the point that saturated pixels start to appear in the images. For instance, although images number 5, 6, 8, and 9 meet both NRFA-FMI criteria, image number 7 has almost the entire tumor area saturated due to the increased camera gain and, therefore, is discarded by the NRFA-FMI method [see Fig. 3(a)].

The BRISQUE and NIQE scores are shown in Figs. 3(c) and 3(d), respectively. Based on a single criterion, unlike the two criteria used in NRFA-FMI, BRISQUE, and NIQE were only able to assign the highest fidelity to the image with the lowest corresponding score. These are image number 21 [Fig. 3(a), orange dotted box] for the BRISQUE method and image number 20 [Fig. 3(a), magenta dotted box]) for the NIQE method. However, both methods have identified "best" images with a substantial number of saturated pixels, and therefore, cannot be considered as high fidelity images.

The importance of the proposed NRFA-FMI method is further highlighted by the histograms depicted in Figs. 3(e)–3(g). These histograms correspond to "best" images defined by each method. For image 9, most of the pixels are concentrated at the lower values of the camera’s dynamic range, which is expected due to the relatively small area of the tumor compared to the large area of the background. In the inset of Fig. 3(e), however, there are pixels distributed across higher values of the camera’s dynamic range due to signals from the mouse body and the tumor area, whereas there are no saturated pixels. On the contrary, the best images chosen by BRISQUE and NIQE have broader peaks that imply higher background and there is considerable saturation as there are ∼50,000 saturated pixels for each method, which corresponds to ∼20% of all image pixels covering the entire body of the mouse as evident from Fig. 3(a).

### 3.4 Expt. 4 – NRFA-FMI for a Moving Target

**Fig. 4:**
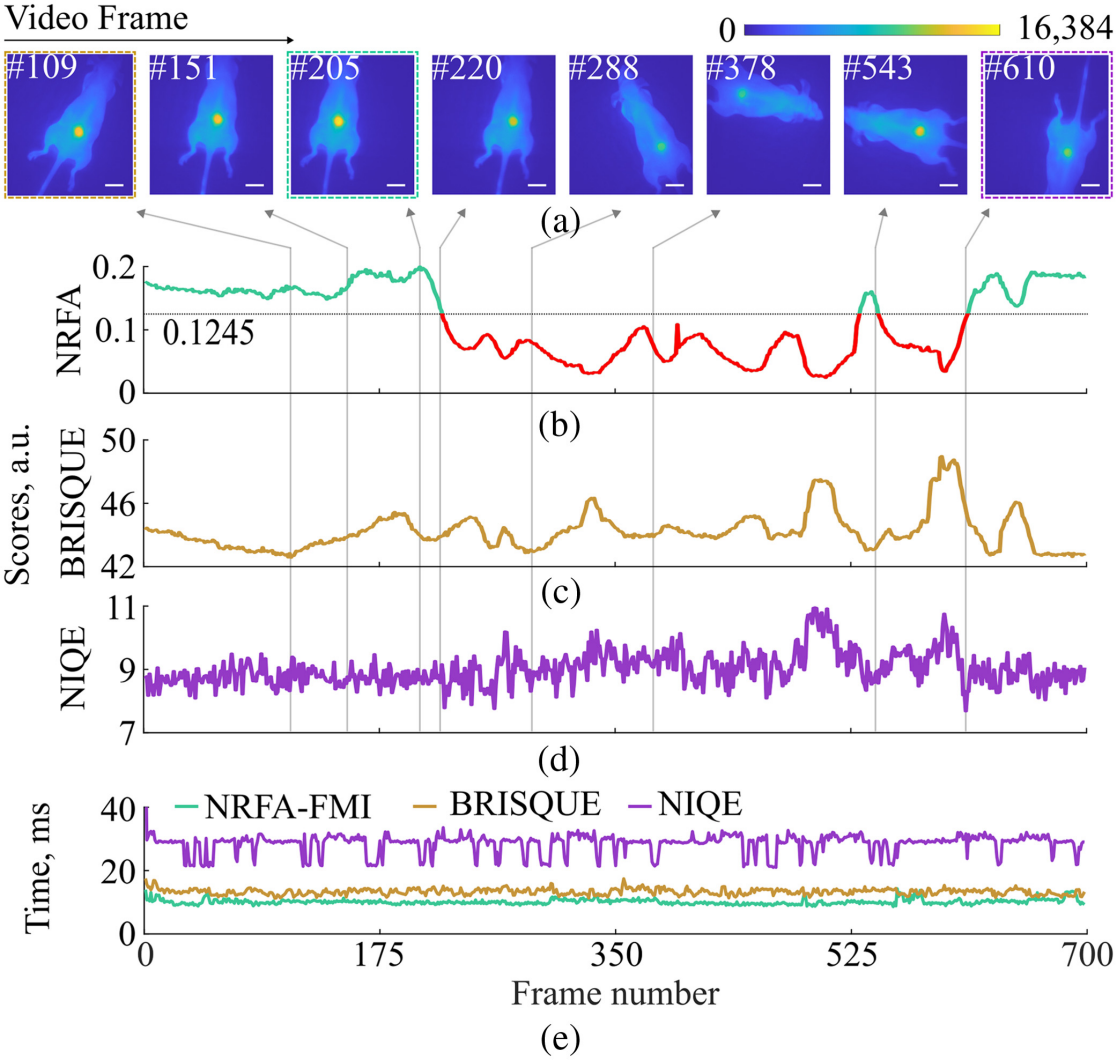
Fidelity assessment of a moving fluorescent target using nonreference fidelity assessment (NRFA-FMI), blind/ referenceless image spatial quality evaluator (BRISQUE) and naturalness image quality evaluator (NIQE) methods. (a) Exemplary frames of the 4T1 tumor mouse model injected with Angiosense 750 at different locations within the FMI system’s field of view. The full range of the acquired frames are shown in Video 1. The scale bars correspond to 2 cm. (b) The ${\mathrm{NRFA}}_{\text{score}}$ for all acquired frames. The NRFA-FMI texture criterion of 0.1245 is marked on the figure (i.e., all scores above this value are marked in green, whereas all scores under the value are marked in red). The frame with the highest ${\mathrm{NRFA}}_{\text{score}}$ is frame #205 [dashed green box in panel (a)]. (c) The BRISQUE scores for all acquired frames. Frame #109 had the lowest score [orange dashed box in panel (a)]. (d) The NIQE scores for all acquired frames. Frame #610 had the lowest score (magenta dashed box in panel (a)). (e) The time required by each method to assess the fidelity of each FMI frame (Video 1, MP4, 5.4 MB [URI: https://doi.org/10.1117/1.JBO.31.6.066001.s1]).

In Expt. 4, the effect of motion and nonuniform illumination on NRFA-FMI performance was examined by imaging the mouse with the 4T1 subcutaneous tumor from Expt. 3 while moving it at different locations within the camera’s FoV. The diffusers used at the system’s illumination port projected a Gaussian-like profile that resulted in the inconsistent readouts during positioning, as shown in the exemplary video frames of Fig. 4(a). The entire sequence of acquired frames is shown in Video 1.

The image fidelity was assessed for all video frames using the NRFA-FMI, BRISQUE, and NIQE methods [Figs. 4(b)–4(d)]. The NRFA-FMI texture criterion for high-fidelity images was 0.1245 and the ${\mathrm{NRFA}}_{\text{score}}$ tended to be above this value when the mouse was in or near the center of the FoV. None of the frames examined had more than 0.1% saturated pixels. These findings are corroborated by visual examination of the exemplary frames shown in Fig. 4(a). To summarize, high-fidelity frames, according the NRFA-FMI method, show a strong contrast between tumor and background. On the contrary, low-fidelity frames had the tumor signal nearly indistinguishable from the background. Video 1 provides additional examples, demonstrating the binary performance (definition of high- or low-fidelity images) of the NRFA-FMI method, as indicated by the green and red colors in Fig. 4(b) and as defined by the method’s two criteria.

By contrast, both BRISQUE and NIQE methods assign scores to the different frames and without any thresholds the distinction between low- and high-fidelity frames seems quite challenging. For example, BRISQUE score for frame #205 in Fig. 4(c) is 43.96 and for frame #378 is 43.87, although there is a clear visual difference between the two frames. Moreover, NIQE scores appear quite noisy, compared with NRFA-FMI and BRISQUE, even though Expt. 4 does not include any rapid changes to the imaging context, making NIQE a quite unreliable model for applications such as FMI.

Finally, we found that the NRFA-FMI method is the fastest method of the three tested in this study, with an average processing time of 10.2±0.8  ms per frame, closely followed by BRISQUE with 13.4±0.9  ms [Fig. 4(e)]. The NIQE method was the slowest at 28.5±3.2  ms per frame. Faster processing speed could be achieved for NRFA-FMI using a real-time programming language, such as C++, instead of MATLAB. Nevertheless, this initial test indicates that the proposed method is faster than other existing methods and, thus, more suitable for integration in the standard clinical practice, where real-time visualization is crucial in FMI applications.

## 4 Discussion

Currently, advanced expertise is needed to determine whether an image is of high quality and representative of the *in vivo* condition to enable accurate interpretation or surgical guidance. With the first clinical applications for FMI showing great promise,^2^^,^^27^^,^^28^ an urgent need exists for real-time, automatic, and unbiased assessment of image fidelity during acquisition. In this study, fluorescence image fidelity is defined operationally as the suitability of an acquired image for reliable interpretation, rather than as similarity to a true underlying fluorophore biodistribution, which is typically inaccessible in realistic FMI settings. Accordingly, NRFA-FMI has been designed as a reference-free screening tool that identifies high- and low-fidelity images based on experimentally grounded surrogate criteria reflecting signal detectability, contrast preservation, resolution, and saturation avoidance. This approach enables practical fidelity assessment without requiring reference images and supports broader clinical acceptance of FMI by reducing subjectivity and minimizing the risk of misinterpretation.

In contrast to other state-of-the-art NR-IQA methods, NRFA-FMI is based on two key criteria that combine multiple metrics indicative of a high-fidelity image. As such, NRFA-FMI can correctly identify high-fidelity images more reliably than the BRISQUE or NIQE methods, which rely on just one score. Importantly, NRFA-FMI identifies all high-fidelity images and not just the best frame out of an image stack. Our NRFA method can be easily adapted to alert nonexpert users when acquired images have insufficient quality and imaging needs to be repeated (see Video 1). These key features of NRFA-FMI demonstrate a great potential for integration into existing FMI applications and systems.

The NRFA-FMI method is based on the combination of histogram and textural statistics as defined by Ref. 1. The main advantage of this approach is that NRFA-FMI does not require manual definition of specific ROIs, which is often user-biased and inconsistent, to quantify performance metrics such as SNR, CNR, TBR, etc. Many recent studies and guidelines still rely on ROI selection for assessing image fidelity and performing quality control of FMI systems. For example, the AAPM recently provided guidelines for the selection of the ROIs for the quantification of SNR and CNR.^4^ Larochelle et al.^29^ also suggested two different approaches for the definition of the ROIs during FMI with the use of anthropomorphic phantoms. Such ROI-based metrics are commonly used for the performance assessment and quality control of FMI systems;^30^^–^^33^ however, they are also suggested as fidelity metrics for clinical and preclinical FMI applications.^34^^,^^35^ Another very common metric in clinical FMI applications is the TBR, which is used to characterize not only the performance of the investigated tracers but also the quality of the acquired data.^34^^,^^36^ However, we have previously demonstrated that the calculation of metrics based on subjectively selected ROIs leads into erratic results^9^ and requires detailed reporting.^37^ By contrast, the NRFA-FMI method utilizes the entire image to assess the quality of an image, making it better suited for fidelity assessment of FMI.

At the same time, the NRFA-FMI method does not require reference images (i.e., a ‘clean’, pristine reference image with respect to which the quality of the acquired image is assessed) for categorizing an image as high or low fidelity. Even with the advent of artificial intelligence, many IQA methods still require a reference image, or ground truth, to assess the fidelity of an image. For example, methods such as qF-SSOP and SAIRC have been used to enhance and assess the quality of fluorescence images,^12^^,^^13^^,^^38^ but these two methods require reference images that are not possible in clinical FMI applications. Another study that used artificial intelligence introduced a generative adversarial network to transform NIR-I or NIR-IIa images to NIR-IIb ones, where SNR and CNR are expected to be maximal.^39^ Despite the promising results, the training of that model and the assessment of the achieved quality still required the presence of reference images. In our opinion, the requirement for reference images in existing methods is a key limitation hindering the full exploitation of artificial intelligence in FMI. By contrast, the NRFA-FMI method can be directly used to assess the fidelity of FMI images in datasets and video streams, therefore, enabling broader and more effective use of artificial intelligence in the field of FMI.

Although the experimental validation in this study focuses on near-infrared fluorescence imaging, NRFA-FMI is not inherently wavelength-specific. Increased autofluorescence, particularly in the visible spectrum, manifests as elevated background signal and reduced contrast, which directly affects histogram utilization and spatial texture. As such, autofluorescence-induced fidelity degradation can be captured by the proposed NRFA-FMI method. Experimental validation of this behavior in visible-wavelength fluorescence imaging represents an important direction for future work.

Image fidelity assessment has advanced most rapidly in the fields of natural imaging and video streaming, where the need for automated quality control is substantial. Such methods, however, are often built and trained to process images containing natural scene statistics, an approach that we show herein to be unsuitable for fluorescent images. The seminal work by Wang et al.^14^ in 2004 was one of the most significant studies in the field of IQA. It introduced the structural similarity index measure (SSIM) between the acquired and reference images, and SSIM has since become a standard in video production and streaming. This work has spurred intense efforts to develop NR-IQA methods most of which are based on perceptual measures that are prevalent and distinct in natural images but are often absent in FMI images. Two such methods, i.e., the BRISQUE^15^ and NIQE^16^ methods were chosen as independent methods for comparing against the NRFA-FMI method’s outcomes. However, both these methods, although widely used in digital imaging, are fundamentally ill-suited for fluorescence imaging due to their reliance on natural scene statistics that are absent in FMI. BRISQUE evaluates locally normalized intensity variations and tends to favor oversaturated fluorescence images, as saturation reduces local variance and artificially aligns image statistics with those of undistorted natural scenes (see Figs. 2 and 3). Conversely, NIQE measures deviations from a fixed natural-image statistical model and therefore preferentially converges to noise-dominated, low-contrast frames, whose smooth intensity distributions more closely resemble its learned priors (see Figs. 1 and 2). NRFA-FMI addresses this challenge by relying exclusively on histogram- and texture-based descriptors that are directly relevant to FMI signal characteristics, explicitly penalizing both saturation and noise-dominated images through independently defined histogram and texture criteria.

A key feature of NRFA-FMI is the definition of two criteria, which were established using an FMI fluorescence standard developed by our group.^20^ Rather than relying on a single scalar score, such as many NR-IQA methods, NRFA-FMI combines complementary histogram- and texture-based descriptors. Specifically, BWF and CLF characterize effective use of detector dynamic range and preservation of image information, whereas the E reflects spatial uniformity and texture degradation associated with insufficient signal or excessive saturation [Sec. 2.4 and Figs. 1(f)–1(h)]. These metrics are combined multiplicatively, ensuring that deficiencies in one critical aspect of image fidelity cannot be compensated by favorable behavior in another. As a result, noise-dominated, oversaturated, or structurally degraded images are consistently identified as low fidelity. An additional advantage is that these criteria are global and do not need to be redefined before each imaging session; for example, the critical values derived during Expt. 1 (Fig. 1) were directly applied in all subsequent experiments in this study.

This global fidelity assessment also facilitates straightforward integration of NRFA-FMI into existing FMI systems and workflows. As shown in Video 1, the NRFA-FMI method can be used to identify and label all low fidelity images and, thus, enable accurate data interpretation, or suggest the need for image re-acquisition or the optimization of system settings for improved readouts.

Finally, the 10.2±0.8  ms that NRFA-FMI needs per frame [see Fig. 4(e)] are significantly faster than the average 80 to 100 fps commonly used in FMI. By employing a real-time oriented programming language, such as C++, and concepts of parallel programming will enable the integration of NRFA-FMI into real-time FMI applications without any observable latency. Although the histogram-based components of NRFA-FMI are normalized to detector bit depth, the absolute fidelity scores and the critical values of the texture and histogram criteria may be influenced by system-specific characteristics such as detector technology, dynamic range, noise behavior, and illumination profile. Nevertheless, the NRFA-FMI framework is generally applicable across FMI systems, with the potential for direct transfer or minimal re-establishment of criteria using standardized measurements. Ongoing and future studies involving markedly different FMI systems will further assess the generalizability of the proposed criteria and their broader adoption.

## 5 Conclusion

This study presents the first implementation of an NR-IQA method tailored to FMI’s requirements. Integrating NRFA-FMI into clinical and preclinical FMI applications will enable more accurate data interpretation and reduce user-imposed bias, which often brings the outcomes of many studies into question. Moreover, by assessing the fidelity of different FMI datasets, NRFA-FMI can potentially support the integration of artificial intelligence into FMI by identifying appropriate training images. Finally, NRFA-FMI’s ability to provide reference-free fidelity assessment makes it a valuable asset for the broad acceptance of FMI as a clinical tool for surgical guidance and diagnosis.

## Supplementary Material

10.1117/1.JBO.31.6.066001.s1

## Data Availability

Data are available from the corresponding author upon reasonable request.
